# Investigating the Impact of Information Sharing in Human Activity Recognition

**DOI:** 10.3390/s22062280

**Published:** 2022-03-16

**Authors:** Muhammad Awais Shafique, Sergi Saurí Marchán

**Affiliations:** 1 Centre Internacional de Mètodes Numèrics en Enginyeria (CIMNE), Universitat Politècnica de Catalunya BarcelonaTech (UPC), 08034 Barcelona, Spain; sergi.sauri@upc.edu; 2 Center for Innovation in Transport (CENIT), Universitat Politècnica de Catalunya BarcelonaTech (UPC), 08034 Barcelona, Spain; 3 Department of Civil Engineering, University of Central Punjab, Lahore 54590, Pakistan

**Keywords:** human activity recognition, machine learning, oversampling, random forest, smartphone

## Abstract

The accuracy of Human Activity Recognition is noticeably affected by the orientation of smartphones during data collection. This study utilized a public domain dataset that was specifically collected to include variations in smartphone positioning. Although the dataset contained records from various sensors, only accelerometer data were used in this study; thus, the developed methodology would preserve smartphone battery and incur low computation costs. A total of 175 different features were extracted from the pre-processed data. Data stratification was conducted in three ways to investigate the effect of information sharing between the training and testing datasets. After data balancing using only the training dataset, ten-fold and LOSO cross-validation were performed using several algorithms, including Support Vector Machine, XGBoost, Random Forest, Naïve Bayes, KNN, and Neural Network. A very simple post-processing algorithm was developed to improve the accuracy. The results reveal that XGBoost takes the least computation time while providing high prediction accuracy. Although Neural Network outperforms XGBoost, XGBoost demonstrates better accuracy with post-processing. The final detection accuracy ranges from 99.8% to 77.6% depending on the level of information sharing. This strongly suggests that when reporting accuracy values, the associated information sharing levels should be provided as well in order to allow the results to be interpreted in the correct context.

## 1. Introduction

Human Activity Recognition (HAR) is a hot research topic today wherein the identification of different human activities is attempted with the help of sensor data [1,2,3]. With the wide array of sensors built into modern smartphones as well as wearable devices such as fitness trackers and smartwatches, the collection of activity-specific data has become very convenient. The understanding of human behaviors by researchers leads to its application in domains such as healthcare, fitness, and home automation [4].

The high penetration rate of smartphones and their profound impact on our daily lives makes them an ideal candidate for context-aware data collection [5]. Sensors such as accelerometers, GPS, gyroscopes, etc., provide a perfect opportunity to infer human activities to an acceptable level using machine learning algorithms, attracting many researchers to this work [6,7,8,9]. Several such studies have reported notable results by deducing information using sensor data [10,11]. However, one aspect to be noted in such studies is that the devices have been carried in a particular manner, mostly attached to various body parts such as the waist or arm. Therefore, their remarkable results may be biased due to the controlled environment used during data collection. Smartphone users cannot be assumed to store their smartphones in a particular position, as smartphones can be stored or held in any orientation deemed comfortable or secure by their owners. Furthermore, they may be in use during traveling, which complicates the activity recognition problem at hand.

While many public domain HAR datasets have been published, most of them are marred by reliance on fixed smartphone positioning and orientation. The UCI HAR dataset was gathered with the help of waist-mounted smartphones, capturing activities such as lying down, sitting, standing, walking, walking downstairs, and walking upstairs [12]. With SVM applied, the study reported higher than 90% detection accuracy. Similarly, another popular HAR dataset collected as part of the WISDM (Wireless Sensor Data Mining) project required respondents to carry their smartphones in the front pockets of their pants while performing various tasks including sitting, standing, walking, jogging, ascending stairs, and descending stairs [13].

In the literature, many studies have been conducted using these datasets. For instance, one study drew a comparison among K-Nearest Neighbors (KNN), Principal Component Analysis (PCA), Random Forest (RF), and Convolutional Neural Networks (CNN) [14]. The study reported that the prediction ability of CNN is better than other algorithms present in the comparison. The paper further investigated the impact of various algorithmic parameters on the optimal settings for CNN. Likewise, another study proposed CNN for addressing the HAR problem [15]. Furthermore, a group of researchers experimented with a deep learning method, the Deep Belief Network (DBN) [16]. They compared and concluded that the performance of the DBN is better than SVM. On the other hand, different studies have explored the relative usefulness of various feature types used by machine learning algorithms [17,18]. The results demonstrated that frequency-domain features perform better than others in exploiting the hidden patterns in data, at least for algorithms such as SVM and CNN.

Moreover, many other studies have collected datasets for research. For example, one group collected data for nine different smartphone orientations when carried in a backpack [19]. The results showed that their developed SVM model outperformed algorithms such as KNN, decision trees, and naïve Bayes. Similar results were reported in other works which incorporated additional sensors, such as GPS and a magnetometer [20,21].

The above discussion shows that while HAR data collected in a controlled environment might yield good prediction results, they are not a true representation of the randomness associated with the data collection methodology. Therefore, this study incorporates a public domain dataset specifically collected to replicate the uncertainty linked to smartphone storage and use during data collection [22]. This dataset takes the methodology one step closer to real-life implementation where the respondents are free to use their smartphones as they please while data are being collected. Of course, this poses a challenge as sensor data cannot be easily analyzed to learn the patterns when the coordinate system keeps changing continuously. Moreover, as the data are collected in an urban setting, traffic congestion may result in confusion while differentiating between walking/jogging and motorized transport. This study takes on the daring task of analyzing such a dataset. Furthermore, the most probable methodology to distinguish between trip and non-trip activity would be to use GPS data, as departure from any point of interest can very easily be recognized. However, GPS has its problems as well. Apart from accuracy issues, privacy concerns and battery use make it difficult to adopt in a real-world application scenario. Under such circumstances, discriminating among various trip and non-trip activities utilizing only accelerometer data seems to be a difficult task. This paper tries to solve that very problem. Moreover, the activity recognition methodology presented in this paper is developed stepwise such that it provides useful insights to readers. Understanding how the algorithm works is key to achieving good results, which is one of the aims of this study. Another aim is to understand how training and testing datasets should be formed; this has a profound impact on detection accuracy, as is demonstrated in this study.

The key contributions of this study can be summarized as follows:

- It takes on the challenging task of analyzing a dataset that is both realistic and difficult to investigate
- The developed methodology relies only on accelerometer data, which reduces data collection and computation costs at the price of accuracy; this study tries to decrease this loss in accuracy
- Various machine-learning algorithms reported in the literature are compared based on their performance metrics, including computation time
- A comparative analysis of various methods in which data can be stratified is provided
- This paper establishes the data sharing level as a key variable to be provided when classification results are reported
- A simple post-processing method is developed that can significantly improve detection accuracy
- The study develops a low-cost methodology for Human Activity Recognition.

## 2. Proposed Methodology

**Figure 1:**
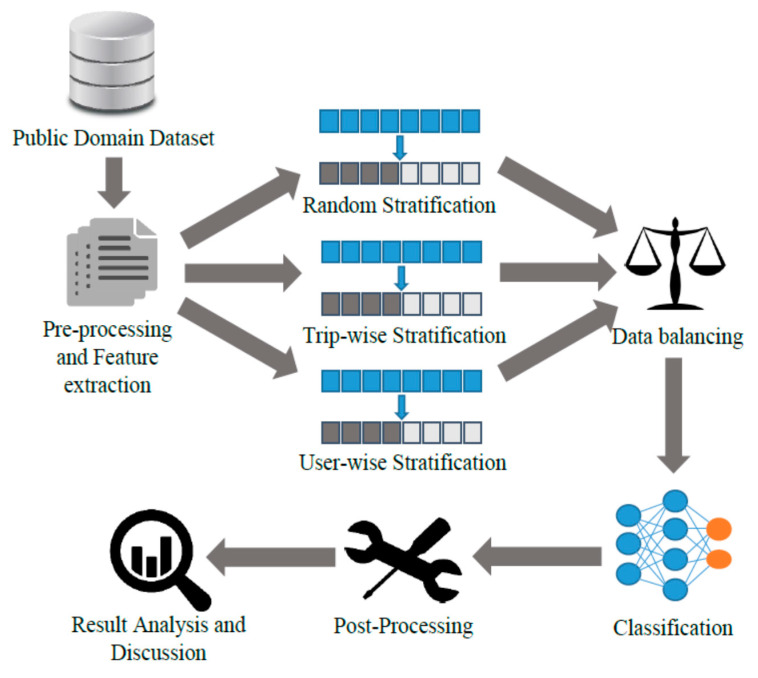
Proposed Methodology of the study.

The proposed approach comprises pre-processing and feature extraction, data stratification, data balancing, classification, post-processing, and results analysis. We began by accessing a public domain dataset for human activity recognition. The data were explored and pre-processed before various features were extracted. To understand the impact of information sharing between training and testing datasets, three types of stratifications were performed, i.e., random, trip-wise, and user-wise. Different data balancing approaches were used for the training data only. This ensured that we had imbalanced test data to predict. Classification was conducted using various supervised learning algorithms. Where required, post-processing was performed in order to improve classification accuracy. Lastly, the results were investigated and conclusions were drawn. Figure 1 summarizes the proposed methodology.

## 3. Pre-Processing and Feature Extraction

### 3.1. Study Data

The database used in this study is publicly available, and its collection process has already been discussed in great detail [22]. This study only utilized the raw accelerometer data present within the cited database. The reason for dropping the remaining sensor data was to make the methodology simpler. Only one sensor’s data means less data to collect and analyze. This saves smartphone batteries from draining too quickly and lowers computation costs. However, the detection accuracy is be compromised.

The sensor data was collected by 18 respondents capturing four activities, i.e., inactive, active, walking and driving. “Inactive” and “active” both correspond to being confined within a single point of interest and not travelling somewhere. The difference, however, stems from the placement of the smartphone. If it is not carried by the respondent and is placed somewhere, such as on the desk while the participant performs various tasks without travelling to a different place such as cooking, shopping, or cleaning, it is classified as “inactive”. On the contrary, if such tasks are performed while the smartphone is carried by the individual, then it is recorded as “active”. Further, “walking” mode includes jogging and running, and “driving” mode means travelling via any motorized means including a car, motorbike, bus, train, etc.

### 3.2. Pre-Processing of Data

The data showed varied levels of collection frequencies among different participants; therefore, the entire dataset was scaled down to 1 Hz. Other than incorporating uniformity in the data, the scaling down reduced the amount of data to be analyzed while retaining the scale of the information to an extent. This step was directly linked to the one of the objectives of this study, i.e., a low computation cost.

The data collection process was unique, as both the trips and sensor data pertaining to activities performed without travelling to a new destination were recorded, and collected. Due to this aspect, activities with fewer intervals were required to be included in the analysis. This posed a computational challenge, as the smaller duration meant smaller window sizes to be used for feature extraction. Nevertheless, a threshold of 30 s was selected, which implied excluding seven activities out of a total of 341 from the dataset.

### 3.3. Feature Extraction

One primary base value and three secondary base values were calculated to start with, as follows (Equations (1) and (2)):

(1) $$Resultant\;Acc,\;acc_{R}=\sqrt{acc_{x}{}^{2}+acc_{y}{}^{2}+acc_{z}{}^{2}}$$

(2) Direction Cosine {cx=accxaccRcy=accyaccRcz=acczaccR

**Figure 2:**
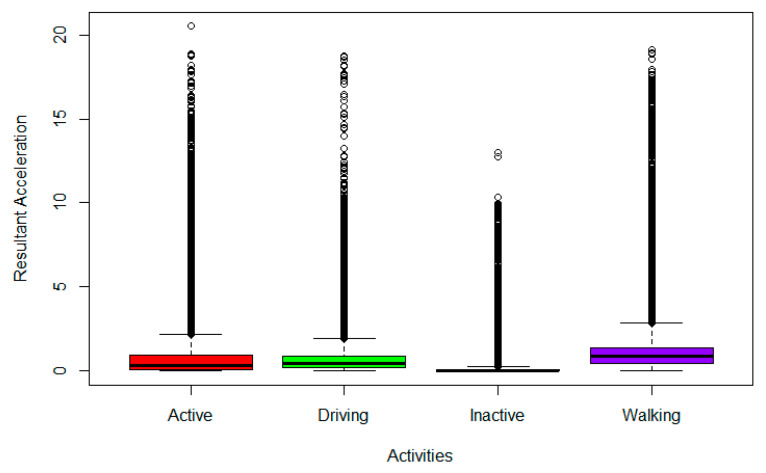
Detection of Outliers.

**Figure 3:**
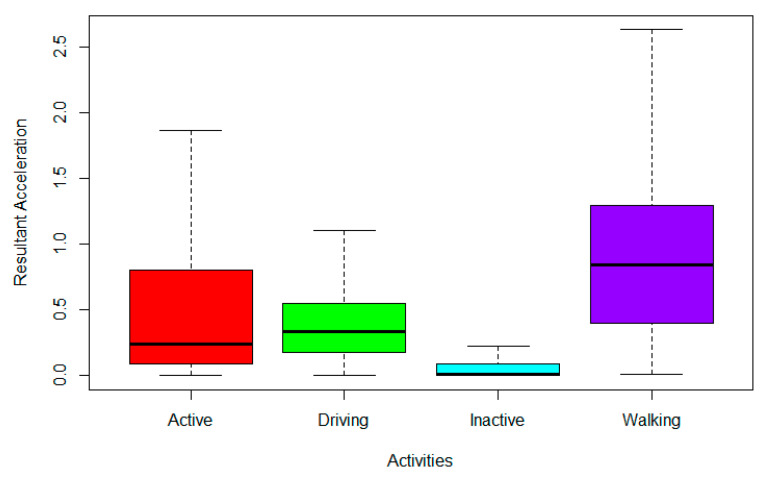
Removal of Outliers.

Using the calculated resultant acceleration (primary base value), outliers were identified (Figure 2) for the observed and removed activities (Figure 3). Other features, including average, maximum, minimum, standard deviation, skewness, kurtosis, and percentiles (5%, 10%, …, 90%, 95%) were extracted from each of the four base values, resulting in 175 features. Various sliding window sizes and overlap values were experimented with for this extraction process, as discussed in the next section.

### 3.4. Window Size and Overlap

**Figure 4:**
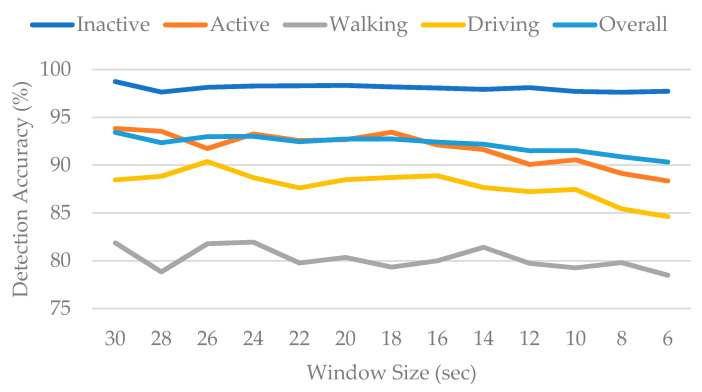
Detection accuracy with respect to window size.

Initially, window sizes ranging from 30 s to 6 s were experimented with, with a 50% overlap. A total of 90% of the data were randomly selected to train the algorithm, while prediction was performed using the remaining 10% of the data. The results, as depicted in Figure 4, reveal that the activity type “Inactive” was predicted with relatively high accuracy, whereas the activity type “Walking” provided the lowest accuracy. Another observation was that the accuracy generally continued to decrease as the window size was reduced. Next, the impact of overlap was studied. For this purpose, three values, i.e., 25%, 50%, and 75% overlap, were experimented with, the results of which are summarized in Table 1. The results suggested that 75% overlap provided better accuracy than the other two; hence, a 28 s sliding window with 75% overlap was used for the final feature extraction.

### 3.5. Amount of Data

The final distribution of the data after pre-processing, cleaning, and feature extraction is shown in Table 2.

## 4. Data Stratification

Three types of data stratifications were performed to investigate the effect of varying levels of information sharing on prediction accuracy.

### 4.1. Random Stratification

The data were randomly stratified into ten parts based on the activities. Hence, each part would have a 10% contribution from the data linked to each activity. As the data points could not be divided equally among the ten parts, the tenth part consequently ended up having slightly more or less data for each activity compared to the other nine parts. Ten-fold cross-validation was performed.

### 4.2. Trip-Wise Stratification

**Figure 5:**
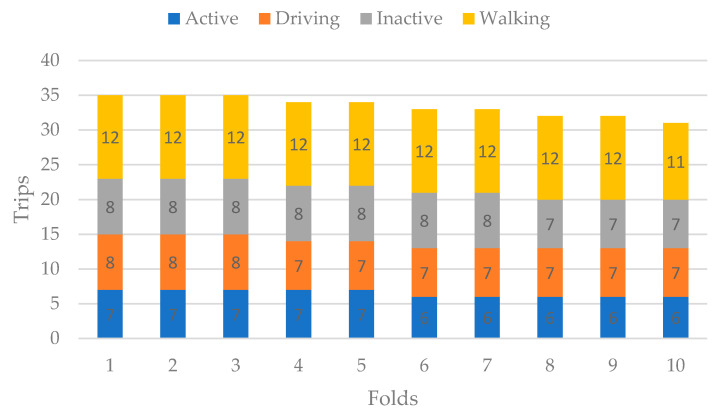
Distribution of trips among ten folds.

The data contained 334 total trips comprising 65 active, 77 inactive, 119 walking, and 73 driving. These numbers were divided among ten folds, as shown in Figure 5. For each fold, the number of trips pertaining to each activity were randomly selected without any consideration of the amount of data within each trip. This led to increased unbalancing among the activities. Again, ten-fold cross-validation was performed.

### 4.3. User-Wise Stratification

Leave One Subject Out (LOSO) cross-validation was performed for these data; however, as several of the participants skipped one or more of the activities appropriate bands were developed for this purpose, as shown in Table 3.

## 5. Data Balancing

Four different methods were applied to balance the data, as follows:

1. Downsampling: A number of samples equal to the minority class were randomly selected from the majority classes
2. Oversampling: A number of duplications equal to the majority class were randomly performed for the minority classes
3. Oversampling and Downsampling: Oversampling of minority classes and downsampling of majority classes was performed to reach the mean value
4. SMOTE: Synthetic Minority Oversampling Technique was investigated

It is worthwhile to note here that unlike many studies where data balancing is performed before dividing the data into training and testing datasets, this study balanced only the training data, which is more realistic.

## 6. Classification

Various machine learning algorithms were used to predict the activities included in the data, including Support Vector Machine (SVM) [22,23], naïve Bayes (NB) [24,25], K-Nearest Neighbor (KNN) [24,26], Random Forest (RF) [27,28], and Extreme Gradient Boosting (XGBoost) [29,30]. The best-performing algorithm was later compared with Feed-forward Neural Network (NN) [31]. These algorithms were selected based on their extensive use in similar studies. A grid-wise analysis was performed for each algorithm in order to identify the optimum values of the associated parameters.

## 7. Post-Processing

To further improve detection accuracy, a post-processing algorithm inspired by the simple method reported by [27,32] was developed. Within each trip, a voting sequence was generated whereby if the predicted value for the *i*-th instance is, for example, “walking”, then one additional vote would be added to walking, and at the same time one vote each would be deducted from the other three activities. This way, a matrix with rows = nrows (test) and columns = 4 would be generated. The maximum vote activity for each instance (row) would then be determined as the final prediction. The algorithm consisted of three voting sequences. First, a forward sequence was carried out initiating from the start of each trip to its end, followed by a backward sequence, and ending with a second forward sequence. This is explained further along with an example in Table 4.

## 8. Evaluation and Analysis

### 8.1. Evaluation Measures

**Figure 6:**
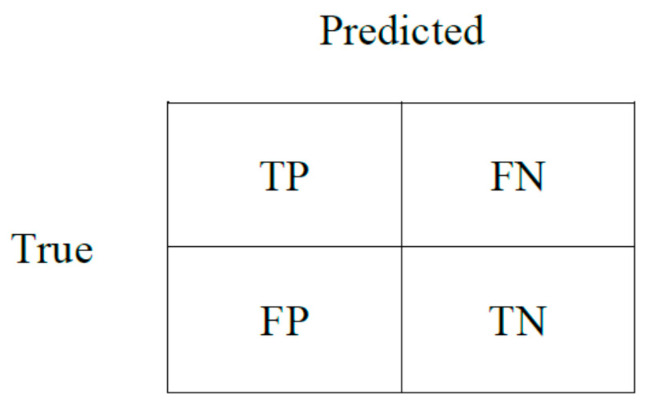
Confusion Matrix.

The evaluation measures used in this study are provided as follows (Equations (3)–(6)), supported by Figure 6.

(3) $$\mathrm{Precision}=\frac{\mathrm{TP}}{\mathrm{TP}+\mathrm{FP}}$$

(4) $$\mathrm{Recall}=\frac{\mathrm{TP}}{\mathrm{TP}+\mathrm{FN}}$$

(5) $$\mathrm{Accuracy}=\frac{\mathrm{TP}+\mathrm{TN}}{\mathrm{TP}+\mathrm{TN}+\mathrm{FP}+\mathrm{FN}}$$

(6) $$F-\mathrm{Score}=2\times \frac{\mathrm{Precision}\;\times \;\mathrm{Recall}}{\mathrm{Precision}+\mathrm{Recall}}$$

### 8.2. Random Stratification Results

The classification results for random stratified data are shown in Table 5. The maximum values are marked as bold. Values within brackets supply standard deviations.

It is evident that XGBoost provides better results most of the time. However, these measures are not enough to draw a solid conclusion. The accuracy and computation time for each algorithm are provided in Table 6. This table completes the picture; it can be seen that SVM closely follows XGB, which in turn is closely followed by RF. Nevertheless, the computation times vary greatly. XGB performs the computation in 2.64 min, whereas for RF and SVM the computation time increases by more than 750% and 7500%, respectively. As this study has as one objective to develop a low-cost methodology, XGB was selected to proceed further.

Next, the effect of data balancing was investigated. Table 7 provides accuracy results by applying various data balancing methods, as discussed in Section 5. From the table, it is clear that a simple oversampling method performs slightly better than SMOTE, and is much quicker.

### 8.3. Trip-Wise Stratification Results

Trip-wise stratification results are provided in Table 8. The results reveal that no substantial improvement is obtained by balancing the training datasets. Nevertheless, downsampling was adopted because it could yield comparable accuracy to others while the amount of training data could be decreased, reducing the overall cost. One important aspect to note here is that the accuracy with trip-wise stratification (87.7%) is significantly lower than that achieved by random stratification (99.8%).

### 8.4. User-Wise Stratification Results

The results yielded by user-wise stratification, the final step in this comparative study, are shown in Table 9. Here, a comparison is made with the state-of-the-art Forward-feed Neural Network. The Neural Network results in better accuracy compared to XGBoost; however, when post-processing is applied after XGBoost, its accuracy experiences a considerable jump, slightly surpassing the Neural Network. In terms of computation time, XGBoost with postprocessing has an even greater edge.

## 9. Discussion

### 9.1. Window Size and Overlap

While a larger window size results in a smoother dataset, it causes the data points to be reduced. However, a smaller window size produces a larger dataset that is more sensitive to variations in data trends. This is the reason for the overfitting of the algorithm for shorter window sizes that bring about reduced detection accuracy, even though the amount of data is relatively greater. Further, the detection accuracy among the activities is directly dependent upon its share in the parent dataset. Figure 4 suggests that the activity “Inactive” shows the highest accuracy, followed by “Active”, “Driving”, and lastly “Walking”.

This corresponds well with the amount of data provided in Table 2. Hence, all other activities are relatively more misclassified as “Inactive” due to the overlearning of this activity type owing to its huge proportion. This is especially evident from the fact that as the window size continues to decrease, the detection accuracies for all activity types fall except for “Inactive”.

A greater overlap means more data points; this is the reason for increased detection accuracy with the same window size. As each window size was kept constant for the three overlap values tested, the resulting smoothness of the extracted features remained the same. Keeping the extent of detail constant, more data points meant more data for the algorithm to train on, hence making it more efficient.

### 9.2. Classification Results

**Figure 7:**
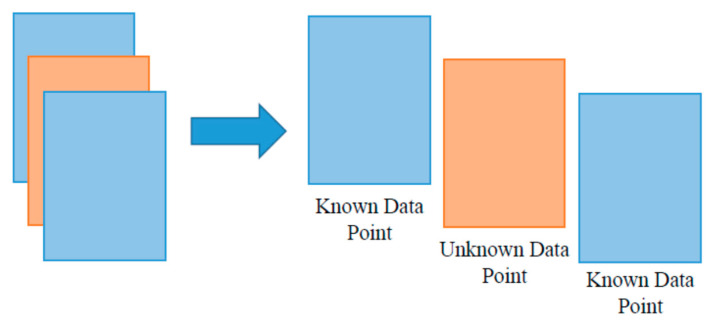
Information sharing at data level.

**Figure 8:**
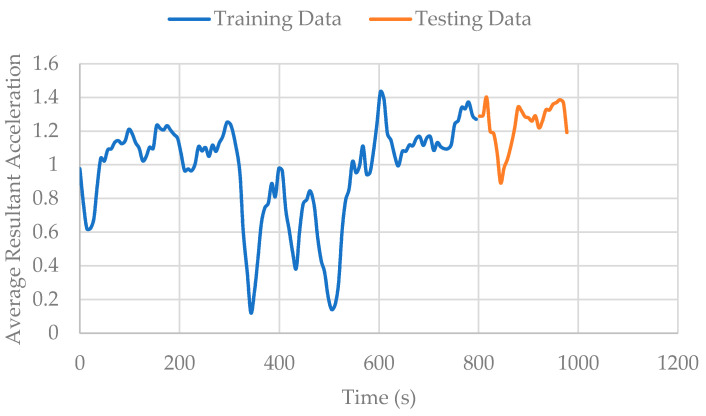
Information sharing at trip level.

**Figure 9:**
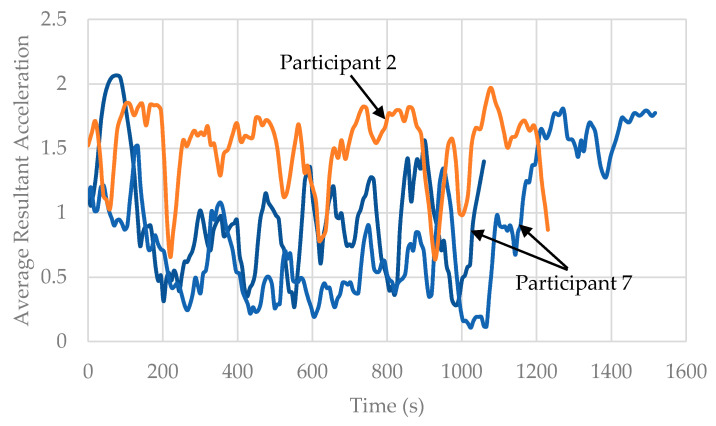
Information sharing at participant level.

The most important observation is that prediction accuracy continues to decrease from 99.8% (random stratification), to 87.7% (trip-wise stratification), to 76.6% (user-wise stratification). The reason behind this drop is the decreasing level of information sharing. In random stratification, information sharing is present at three levels. First, the features are extracted by sliding windows, and every data point shares 75% of information with the previous one as well as providing 75% of information to the next one. Thus, a randomly selected unknown data point can very easily be predicted by its neighboring known points (Figure 7). Second, every individual trip has a specific trend. If a part of that trend is known, there is high probability of accurately predicting the remaining unknown part (Figure 8). Third, each individual participant has predictable movement patterns. Hence, if the algorithm learns a walking trip for a specific participant, it can predict another walking trip for the same individual with relative ease. Figure 9 shows data from two walking trips by Participant 7 and a single walking trip by Participant 2. It is clear from the Figure that an algorithm trained on one of the trips by Participant 7 would predict the other trip by the same participant with relatively ease compared to the trip made by Participant 2. When trip-wise stratification is performed, the first two types of information sharing scenarios are eliminated, and the third scenario of information sharing is removed as well when user-wise stratification is performed.

The relatively moderate final accuracy (user-wise stratification) reported in this study may be due to the following reasons.

- The data used in this study are quite unusual. First, they were not collected in a controlled environment where the smartphone positioning is fixed. This includes greater variability in the data. Second, they cover motorized transport captured in an urban setting. This is challenging, as it is difficult to differentiate between a person jogging and a person in a slow-moving car, especially when smartphone positioning is not fixed. Third, this study only takes into account accelerometer data, in order to make the approach more cost efficient.
- The complete removal of information sharing, which has a considerable impact on detection accuracy.
- Efforts to reduce the overall computation cost of the developed methodology take a toll on the accuracy.

## 10. Conclusions and Future Work

This study provides a low-cost methodology for human activity recognition as well as a comparative analysis of the level of information sharing between training and testing datasets and its impact on the prediction accuracy. Below are the main conclusions that can be drawn from this study:

- A larger window size tends to provide better accuracy; however, due to the limitations of the data used and the need to include non-trip activities, the upper threshold was not detected.
- Greater overlap results in both a greater number of data points and higher information sharing among those data points. This may be the reason for the increased prediction accuracy.
- Among the tested conventional machine-learning algorithms XGBoost outperforms all the others, yielding high prediction accuracy while requiring low computation time.
- Simpler methods of data balancing work equally well when compared with SMOTE, and require a relatively short time for computation.
- Decreasing information sharing between the training and testing datasets drastically decreases accuracy, from 99.8% to 77.6%. Therefore, researchers should report the level of information sharing associated with their results in order to allow them to be interpreted in their proper context.
- The Neural Network demonstrates better prediction accuracy than XGBoost; however, the gap can be closed with a simple post-processing step. More importantly, the computation time is relatively low for XGBoost (3.2 min vs. 14.16 min), making it a better option than Neural Network.

It is expected that cost-efficient deep learning algorithms might be able to improve the classification, and it is intended that such a study shall be conducted in the future.

## Figures and Tables

**Table 1:** Detection accuracy with varying overlap values.

Window Size	Overlap (%)	Detection Accuracy (%)				
		Inactive	Active	Walking	Driving	Overall
28 s	25	97.22	92.13	77.55	84.81	91.16
	50	98.24	93.29	82.78	88.90	93.34
	75	99.02	95.81	87.24	92.92	95.47
24 s	25	98.43	90.67	80.88	86.88	92.19
	50	98.48	92.50	84.27	88.32	93.23
	75	99.07	94.61	87.60	93.66	95.37
20 s	25	98.17	93.17	79.02	87.92	92.57
	50	98.18	92.46	80.76	87.17	92.38
	75	98.56	95.45	88.11	92.91	95.35
16 s	25	97.57	91.01	77.85	85.60	91.03
	50	98.38	91.69	82.03	89.01	92.73
	75	98.66	94.23	86.29	92.51	94.77
12 s	25	97.88	89.43	78.21	85.95	90.89
	50	98.10	90.46	78.47	87.81	91.77
	75	98.63	93.61	85.48	90.45	94.17
8 s	25	97.89	87.87	76.61	84.65	90.09
	50	97.79	90.09	80.08	86.15	91.30
	75	98.60	92.23	84.60	89.86	93.49

**Table 2:** Amount of data with respect to activities.

Activity	No. of Participants	No. of Trips	Amount of Data	Percentage
Inactive	13	77	40,297	45.95
Active	9	65	22,516	25.67
Walking	14	119	12,417	14.16
Driving	12	73	12,471	14.22

**Table 3:** Participant Bands for LOSO cross-validation.

Band	Participant	Inactive	Active	Walking	Driving
1	1	666	0	0	153
	6	0	2008	973	0
2	2	159	0	675	356
	13	1407	495	86	0
3	3	20	876	1014	602
	7	1214	0	906	1869
4	4	849	868	63	260
5	5	331	487	489	3499
	14	152	0	204	0
6	8	29,865	1403	4216	1441
7	9	1724	0	2365	655
	11	1013	12,134	618	1289
8	10	0	1859	279	1885
	15	519	0	45	96
9	12	2378	2386	484	366

**Table 4:** Example of a forward and backward voting sequence.

Trip No.	Actual	Predicted	Forward Voting Sequence	Corrected Prediction	Backward Voting Sequence	Corrected Prediction
1	Walking	Walking	0, 0, 1, 0	Walking	0, 0, 8, 0	Walking
1	Walking	Walking	0, 0, 2, 0	Walking	0, 0, 7, 0	Walking
1	Walking	Walking	0, 0, 3, 0	Walking	0, 0, 6, 0	Walking
1	Walking	Driving	0, 0, 2, 1	Walking	0, 0, 5, 0	Walking
1	Walking	Driving	0, 0, 1, 2	Driving	0, 0, 4, 1	Walking
1	Walking	Walking	0, 0, 2, 1	Walking	0, 0, 5, 0	Walking
1	Walking	Walking	0, 0, 3, 0	Walking	0, 0, 4, 0	Walking
1	Walking	Walking	0, 0, 4, 0	Walking	0, 0, 3, 0	Walking
1	Walking	Walking	0, 0, 5, 0	Walking	0, 0, 2, 0	Walking
1	Walking	Driving	0, 0, 4, 1	Walking	**0, 0, 1, 0**	Walking
2	Inactive	Active	**0, 1, 0, 0**	Active	4, 1, 0, 0	Inactive
2	Inactive	Inactive	1, 0, 0, 0	Inactive	5, 0, 0, 0	Inactive
2	Inactive	Inactive	2, 0, 0, 0	Inactive	4, 0, 0, 0	Inactive
2	Inactive	Inactive	3, 0, 0, 0	Inactive	3, 0, 0, 0	Inactive
2	Inactive	Inactive	4, 0, 0, 0	Inactive	2, 0, 0, 0	Inactive
2	Inactive	Active	3, 1, 0, 0	Inactive	**1, 0, 0, 0**	Inactive

**Table 5:** Classification results for random stratified data.

Algorithm	Measure	Inactive	Active	Walking	Driving
XGB	Precision	**0.978** (0.023)	0.807 (0.135)	0.869 (0.112)	**0.876** (0.066)
	Recall	0.929 (0.111)	**0.877** (0.102)	**0.819** (0.127)	**0.884** (0.065)
	F-Score	0.95 (0.07)	**0.835** (0.107)	**0.84** (0.11)	**0.878** (0.051)
RF	Precision	0.975 (0.029)	0.771 (0.219)	**0.873** (0.107)	0.825 (0.181)
	Recall	0.933 (0.106)	0.818 (0.243)	0.811 (0.141)	0.861 (0.095)
	F-Score	0.951 (0.068)	0.788 (0.224)	0.836 (0.117)	0.835 (0.146)
SVM	Precision	0.959 (0.023)	**0.816** (0.125)	0.868 (0.107)	0.856 (0.07)
	Recall	0.947 (0.097)	0.829 (0.114)	0.816 (0.125)	0.859 (0.093)
	F-Score	0.951 (0.058)	0.814 (0.096)	0.834 (0.1)	0.853 (0.063)
NB	Precision	0.934 (0.063)	0.707 (0.25)	0.703 (0.185)	0.588 (0.142)
	Recall	**0.974** (0.051)	0.476 (0.222)	0.705 (0.275)	0.815 (0.107)
	F-Score	**0.952** (0.049)	0.555 (0.219)	0.685 (0.231)	0.674 (0.115)
KNN	Precision	0.943 (0.024)	0.788 (0.128)	0.781 (0.117)	0.812 (0.09)
	Recall	0.937 (0.108)	0.761 (0.136)	0.781 (0.13)	0.834 (0.088)
	F-Score	0.937 (0.064)	0.766 (0.113)	0.777 (0.116)	0.819 (0.069)

**Table 6:** Accuracy and computation time for randomly stratified data.

Algorithm	Accuracy	Computation Time (min)
XGB	**0.894** (0.075)	2.64
RF	0.876 (0.114)	22.86
SVM	0.886 (0.062)	208.13
NB	0.786 (0.095)	5.85
KNN	0.855 (0.069)	295.02

**Table 7:** Accuracy values after data balancing for randomly stratified data.

Balancing Method	Accuracy
Downsampling	0.983 (0.002)
Oversampling	**0.998** (0.0003)
Both	0.995 (0.001)
SMOTE	0.997 (0.0002)

**Table 8:** Results for trip-wise stratified data.

Balancing Method	Measure	Inactive	Active	Walking	Driving	Accuracy
None	Precision	0.935 (0.105)	0.785 (0.212)	**0.729** (0.2)	**0.8** (0.161)	0.868 (0.084)
	Recall	**0.966** (0.042)	0.677 (0.212)	0.798 (0.101)	**0.913** (0.082)	
	F-Score	0.948 (0.073)	0.693 (0.2)	0.735 (0.127)	**0.846** (0.123)	
Downsampling	Precision	0.961 (0.054)	0.8 (0.231)	0.727 (0.205)	0.793 (0.158)	**0.877** (0.067)
	Recall	0.952 (0.047)	0.694 (0.199)	**0.844** (0.083)	0.909 (0.1)	
	F-Score	0.956 (0.041)	0.709 (0.203)	**0.759** (0.146)	0.841 (0.126)	
Oversampling	Precision	**0.971** (0.034)	**0.84** (0.126)	0.654 (0.135)	0.773 (0.215)	0.871 (0.054)
	Recall	**0.966** (0.035)	**0.726** (0.234)	0.822 (0.151)	0.838 (0.146)	
	F-Score	**0.968** (0.018)	**0.751** (0.173)	0.712 (0.102)	0.775 (0.16)	

**Table 9:** Results for user-wise stratified data.

Algorithm	Measure	Inactive	Active	Walking	Driving	Accuracy	Time (min)
XGB	Precision	0.878 (0.152)	0.68 (0.367)	0.554 (0.254)	0.635 (0.26)	0.695 (0.175)	3.03
	Recall	0.908 (0.172)	0.467 (0.337)	0.791 (0.195)	0.646 (0.281)		
	F-Score	0.878 (0.148)	0.486 (0.279)	0.59 (0.202)	0.563 (0.244)		
NN	Precision	0.697 (0.275)	0.62 (0.238)	0.62 (0.231)	0.814 (0.171)	0.731 (0.091)	14.16
	Recall	0.711 (0.228)	**0.609** (0.275)	0.763 (0.204)	**0.848** (0.142)		
	F-Score	0.639 (0.208)	0.529 (0.232)	0.649 (0.205)	**0.819** (0.136)		
XGB with Postprocessing	Precision	**0.969** (0.06)	**0.81** (0.311)	**0.675** (0.355)	**0.836** (0.274)	**0.766** (0.21)	3.2
	Recall	**0.975** (0.047)	0.508 (0.45)	**0.858** (0.21)	0.772 (0.274)		
	F-Score	**0.97** (0.04)	**0.665** (0.324)	**0.66** (0.296)	0.744 (0.357)		

## Data Availability

All data used during the study are available online (Garcia-Gonzalez, D.; Rivero, D.; Fernandez-Blanco, E.; Luaces, M.R. A Public Domain Dataset for Real-Life Human Activity Recognition Using Smartphone Sensors. Available online: https://data.mendeley.com/datasets/3xm88g6m6d/2 (accessed on 21 April 2021)).
